# High prevalence of GII norovirus in hospitalized children with acute diarrhea, in Beijing

**DOI:** 10.1371/journal.pone.0179839

**Published:** 2017-06-29

**Authors:** Liping Jia, You Zhang, Liying Liu, Huijin Dong, Linqing Zhao, Yuan Qian

**Affiliations:** Beijing Key Laboratory of Etiology of Viral Disease in Children, Laboratory of Virology, Capital Institute of Pediatrics, Beijing, China; University of Hong Kong, HONG KONG

## Abstract

This study was addressed to the relationship between norovirus and acute diarrhea in hospitalized children, including hospital-acquired infection (HAI) and community-acquired infection (CAI) in a children's hospital in Beijing. RT-PCR was used to detect norovirus in stool specimen, followed by sequence analysis for PCR products. From 2010 to 2013, a total of 1248 specimens, including 661 from the HAI group and 587 from the CAI group were tested for norovirus. Norovirus were detected in 380 of 1248 (30.4%) diarrheal specimens. The positive rate for norovirus detection was higher in children within HAI group than CAI group (35.3%, 232/661 vs. 25.6%, 148/587), and the difference was significant (*X*^2^ = 14.35, P<0.05). For age distribution, the highest positivity rates of norovirus were in age of 0–5 months for HAI group and 12–23 months for CAI group. In the study, 262 amplicons of the VP1 region from norovirus-positive specimens were sequenced, which showed GII.3 and GII.4 norovirus were the most common genotypes detected in 50.0% (n = 131) and 48.9% (n = 128) of the positive specimens, respectively. Regarding the wards distribution, GII.3 norovirus was mainly detected in ward for neonatal diseases (36/85 in HAI group; 19/46 in CAI group), GII.4 norovirus was mainly detected in ward for respiratory and digestive diseases (21/85 in HAI group; 15/33 in CAI group). Conclusion: The data elaborated the importance of norovirus in hospital associated infectious diarrhea. The prevalence of norovirus is higher from HAI group than CAI group, and the norovirus from the patients in CAI group could be the source of infection in HAI group.

## Introduction

Acute diarrhea is one of the most important causes of high morbidity and mortality in infants and young children worldwide. Viral agents known to cause acute diarrhea include rotavirus, calicivirus, adenovirus and astrovirus [1,2]. Among them, norovirus used to be called Norwalk virus within the family *Caliciviridae* was the first recognized viral agent causing acute gastroenteritis in humans [3,4]. Moreover, with developing of molecular techniques for virus detection, the importance of the role of norovirus in acute diarrhea has been better appreciated and norovirus has become the leading cause of acute gastroenteritis in all age-groups [5–7].

As a member of the *Caliciviridae* family, norovirus is a single-stranded RNA, non-enveloped virus with icosahedral symmetry, and is known to be spread through person-to-person, contaminated food and water, and maybe also through vomits and airborne transmission [4,8,9]. Moreover, norovirus is extremely easy to cause infection, less than 100 virions needed to cause infection [10], and is highly resistant to conventional cleaning agents and long lasting virus shedding from a few days to several weeks [11]. Based on the characteristics mentioned above, control for norovirus spreading is quite difficult. Therefore, people in closed settings like cruise ships, nursing homes, recreational area camps, military facilities, schools, hospitals, and restaurants are more susceptible to be infected. Norovirus infection has generally an incubation period of 24~48 hrs [12] and is characterized by acute onset of nausea, vomiting, abdominal cramps, myalgia, and non-bloody diarrhea.

Acute diarrhea due to norovirus infection has been better recognized in young children in recent years. However, there was little data available on the role of norovirus in hospitalized children both with community-acquired infections (CAI) and hospital-acquired infections (HAI) [13–17], because the lack of active surveillance for norovirus infections among hospitalized patients. Therefore, an investigation was carried out in pediatric patients who developed acute diarrhea during the period of hospitalization in a children’s hospital in Beijing, from 2010 to 2013. This study was to further elaborate the role of norovirus in hospital-associated infectious diarrhea and related risks in general wards which mean not specialized ward for gastroenteritis in children’s hospital.

## Materials and methods

### Study population

The study was carried out at the Affiliated Children’s Hospital of the Capital Institute of Pediatrics in Beijing. As a tertiary hospital, this hospital serves about 1.5 million outpatients and 13,000 inpatients a year and has a total of 410 inpatient beds divided into 7 wards. The ward 1 ~ 4 and 7 are for the patients admitted mainly for neonatal diseases (ward 1), respiratory and digestive diseases (ward 2), neurological and endocrinology disorders (ward 3), cardiovascular and renal disease (ward 4), hematopoietic system or immune system disease (ward 7). Patients who needed surgery were admitted in the ward 5 and ward.Acute diarrhea was defined as 3 loose or looser-than-normal stools in a 24-hour period and/or significant changes to the fecal exterior, excluding the presence of pus or blood. For this study, enrolled hospitalized children were divided into two groups. Children who developed acute diarrhea within 48 hours of hospital admission were assigned to CAI group. This group includes children hospitalized due to diarrhea and children hospitalized due to other cause and developed diarrhea within the first 48 hours of admission. Children whose symptom of acute diarrhea occurred after 48 hours from admission were assigned into HAI group [18]. Clinical-epidemiological variables studied such as gender, age and symptoms, were obtained by reviewing hospital admission records. As a retrospective study, hospitalized children under 15 years of age with onsets of acute diarrhea and meet the above criteria were enrolled in this study during January 2010 and December 2013.

The study was approved by the Ethics Committee of the Capital Institute of Pediatrics. Fecal specimens used for norovirus screening for this study were the remnant after routine tests, so informed consent was waived for participants.

### Virological studies

#### Fecal specimens

In this hospital, fecal specimens collected from children with acute diarrhea were routinely examined for rotavirus using the Rotascreen®Dipstick (Beijing WanTai Biological Pharmacy Enterprise Co. Ltd., Beijing, China) and for adenovirus using polymerase chain reaction (PCR) developed in this laboratory as published previously [19]. After routine tests fecal specimens were stored at -20°C for further analysis.

#### Viral RNA extraction

Fecal samples were diluted 1:10 in ddH_2_O (20 ul/180 ul), followed by centrifuging at 5,000 rpm for 5 min. The supernatants were removed to sterile tubes for viral nucleic acids extraction with TRIzol Reagent (Invitrogen, USA) by following the manufacturer’s instructions. The extracted RNA was re-suspended in 20 ul of RNase-free ddH2O and used in reverse transcription polymerase chain reaction (RT-PCR).

### Detection of norovirus

Random primer was used in the reaction of reverse transcription, 5 ul extracted nucleic acid was first incubated with 500 mM dNTPs and 50 ng random primers (Invitrogen) at 65°C for 5 min, then to reverse transcription following the instructions of MLV (Invitrogen). Then the reverse transcribed cDNA was used in PCR to detect norovirus. The primer sets GISKF/GISKR and GIISKF/GIISKR were used to amplify the capsid encoding genes of GI and GII norovirus with the expected amplicons of 329 and 343 bp respectively [20]. PCR was performed at 95°C for 5 min followed by 35 cycles at 94°C for 30 s, 55°C for 30 s, and 72°C for 90 s, and then a last extension step at 72°C for 10 min. Positive control and negative controls were included in those procedures. The PCR products were identified by 2% agarose gel electrophoresis.

#### Sequencing, genotyping and phylogenetic analyzing

PCR products from norovirus positive fecal samples were purified and then sequenced with corresponding primers mentioned above by Life Technologies of Invitrogen, China. The genotype of the detected norovirus was first determined by the web-based norovirus typing tool v.1.0 (http://www.rivm.nl/mpf/norovirus/typingtool) [21]. To further confirm, nucleotide sequences were phylogenetically analyzed along with the sequences from reference strains available in the GenBank database. MEGA version 5.0 was used for phylogenetic analysis. Sequence alignments were performed with Clustal W and the phylogenetic tree was inferred using the neighbor-joining method. Bootstrap analysis with 1000 pseudo-replicate data sets was used for the statistical significance of the phylogenies constructed. The sequences have been deposited in the GenBank database, and the accession numbers are KY594416 ~ KY594674.

### Statistical analysis

Data analysis was performed with SPSS software version 19.0. Chi-square (*X*^2^) tests were carried out and relationship were considered significant when the adjusted *p* value was less than 0.05.

## Result

During the study period, a total of 3078 fecal specimens were collected from hospitalized children with acute diarrhea. The detection rate of rotavirus and adenovirus were 22.0% and 10.3% respectively. Of the 3078 stool specimens collected for this study, 1248 (40.5%) stool samples were used for norovirus testing, including 661 (53.0%) from the HAI group and 587 (47.0%) from the CAI group.

The different detection rates of norovirus between the HAI group and CAI group were shown in S1 Table. In general, norovirus were detected in 380 of 1248 (30.4%) diarrheal specimens. Norovirus/rotavirus co-infection was found in 42 of 380 specimens, norovirus/adenovirus co-infection was found in 31 of 380 specimens, and rotavirus/adenovirus/norovirus co-infection was in 3 of 380 specimens. The positive rate of norovirus in the HAI group was 35.3% (232/661), while the positive rate of the CAI group was 25.6% (148/587) (X^2^ = 14.35 *p* < 0.05).

The samples collected from each year and seasonal distribution of those norovirus positive during the study period are indicated in S1 Table and S1 Fig. During the study period, norovirus was detected throughout the years, while for the HAI group, norovirus was absent in May 2012, Jun 2013, and for CAI group, norovirus was absent in Jan 2011. Peak of norovirus detection from CAI group was in Aug 2010 (57.1%), Oct 2011 (40.9%), Oct 2012 (52.6%) and Apr 2013 (50.0%), and from HAI group was in Sep 2010 (62.5%), Oct 2011 (54.3%), Oct 2012 (58.3%) and Apr 2013 (50.0%), indicating that the peaks of norovirus infection were consistent between CAI group and HAI group. As shown in S1 Table, the detection rate of norovirus in HAI group was significantly higher than that of the CAI group in 2011 (X^2^ = 9.81, *p* <0.05). Although the positivity rate of norovirus of HAI group was higher than that of CAI group in 2010, 2012 and 2013, the differences turned out to be statistically insignificant (*p* > 0.05).

Specimens were collected from 795 boys and 453 girls. The age range of the enrolled children with acute diarrhea described above was between 3 days and 15 years. When the children with positive samples were divided into 5 age groups (S1 Table), it was shown that the positivity rate of norovirus for HAI group was higher than CAI group, whether for boys (X^2^ = 7.38, *p* <0.05) or girls (X^2^ = 5.95, *p* <0.05). Within each age group, the positivity rate of norovirus from HAI was higher than for CAI group in age of 0–5 months (Girl, X^2^ = 10.94, *p* <0.05; Boy X^2^ = 5.18, *p* <0.05). Regarding the age distributions of girls, the highest rates of norovirus-associated diarrhea were in age of 0–5 month for HAI group and 12–23 month for CAI group. And the difference between age groups was insignificant (HAI, *X*^*2*^ = 5.12, *p*>0.05; CAI, *X*^*2*^ = 8.62, *p*>0.05). For the age distributions of boys, the highest rates of norovirus-associated diarrhea were in age of 12–23 months for both HAI group and CAI group. But difference between age groups of boy was significant (HAI, *X*^*2*^ = 17.20, *p*<0.05; CAI, *X*^*2*^ = 12.23, *p*<0.05).

Distributions of norovirus detection in different wards were shown in S1 Table, the detection rates of norovirus for HAI group were higher than that of CAI group in all wards, except in surgical wards (wards 5 and 6), but the difference of detection rate within HAI groups and CAI groups between wards was significant only in the ward 1 (*X*^2^ = 9.27, *p* <0.05) and ward 2 (*X*^2^ = 8.99, *p* <0.05).

All the positive samples (n = 380) were detected by using primers targeting genogroup GII but not GI. And 262 amplicons of the partial VP1 region from norovirus-positive specimens were sequenced and the genotypes for them were first determined by the web-based norovirus typing tool v.1.0. Of these sequences, 163 belong to the HAI group and the 99 belong to the CAI group. The sequences from the two groups were phylogenetic analyzed, respectively. Among these strains, three genotypes were identified, including genotypes GII.4, GII.3 and GII.13 and the homology of nucleotide sequence is high between strains from the same ward (S2 Fig). Genotypes GII.3 and GII.4 were the most common genotypes detected in 131 cases (50.0%) and 128 cases (48.9%), respectively, and 3 strains were belong to GII.13. Among these 128 GII.4 strains, variants GII.4 Den Haag_2006b, GII.4 New Orleans_2009 and GII.4 Sydney_2012 were account for 71.1% (n = 91), 4.7% (n = 6) and 24.2% (n = 31), respectively. The distribution of genotypes was shown in S2 Table, for both groups HAI and CAI, distribution of GII.4 Den Haag_2006b was in 2010 to 2012, GII.4 Sydney_2012 emerged in 2012 and prevalent in 2013 and no Den Haag_2006b was detected in 2013. For the wards distribution, GII.3 norovirus mainly detected in ward 1 (36/85 in HAI group; 19/46 in CAI group), GII.4 norovirus mainly detected in ward 2 (21/85 in HAI group; 15/33 in CAI group).

## Discussion

The data from this study presented a detailed description for the infection by norovirus in hospitalized children with acute diarrhea, including both HAI group and CAI group. In this children’s hospital affiliated to Capital Institute of Pediatrics, rotavirus and adenovirus were tested routinely for hospitalized patients with acute diarrhea. The positivity rate of rotavirus infection was 22% by antigen detection and adenovirus was 10.3% by PCR, respectively. However the positivity rate of norovirus was 30.4% during the study period, which was higher than that of rotavirus and adenovirus in the same hospital, suggested that norovirus has become the most frequent cause of acute diarrhea in hospitalized children.

The result shows that the detection rate of norovirus from cases of CAI group and HAI group was different significantly. It has been reported that norovirus is a common cause of nosocomial infection in the immunocompromised, the elderly, and newborns [22–24]. Thus far, very few reports in literature compared the norovirus infection between CAI group and HAI group in children's hospital, especially in China. One article in *The Medical Journal of Australia* pointed out that hospital-acquired infections in infants and children need to be paid attention to [25]. It had been reported in the article that 33% of norovirus infection of the inpatients was hospital-acquired. This is very similar to the results of our study. Beersma et al found that the proportion of hospital-acquired norovirus infection was 59% in the youngest patients [22]. In Denmark, the majority of norovirus infections in hospitalized patients were nosocomial, and 28% of the nosocomial infections were in children and adolescents (age <18 years) [23]. In the German Surveillance System, nosocomial infections are also particularly prevalent in the very young [26]. Whereas for CAI group in this study, the rate of norovirus detection was 25.6% which are consistent with some of the other reports with clearly mentioned specimen sources, such as 26% in Finland, 23% in Hong Kong, 27% in Spain [14,27,28]. There are also some lower positivity rates of norovirus from the CAI group, for example 10.4% in North Italy, 8.3% in Paris, 8.5% in United States [15,17,29]. Overall, the positivity rate of norovirus in HAI group children is obviously higher than that of CAI group, demonstrated that nosocomial infection could not be neglected, because this may make treatment for patients further complicated, so active approach to prevent and control HAI by norovirus may effectively reduce the patient's economic burden. Moreover, Compare with the outpatient children with acute diarrhea in Beijing [30], the positivity rate for norovirus in outpatients in the same area were significantly lower than either the CAI group or the HAI group of norovirus infection in hospitalized children. Norovirus is highly stable in the environment and resistant to conventional cleaning agents, norovirus were also detected in inanimate surfaces [31]. In addition, hospital is a closed setting, so active monitoring for norovirus infections in hospitalized patients is necessary.

There are some differences in seasonal distribution between groups of HAI and CAI. For the CAI group, the norovirus activities were more common in winter. However, no obvious seasonal distribution of norovirus in the HAI group was shown, which may attribute to hospital-acquired infection and the susceptibility of the sick children.

For each year, the detected rates of norovirus for HAI group was higher than that of CAI group, but the result turned out to be statistically significant only in 2011. Considering the number of selected specimens, some bias may exist, as more sample were collected in 2011. The highest prevalence of norovirus was seen among young children and in ward 1 and ward 2. Those with neonatal diseases were admitted in ward 1. It is known that new born baby within neonatal period has the highest morbidity and mortality. The high susceptibility of norovirus in the very young may also due to hygienic conditions, because all the little babies should wear diapers. During changing diapers, the hands of healthcareres maybe an important transmission route of the norovirus infection. In ward 2, there still has high prevalence of norovirus infection which may due to the admission of digestive diseases including acute gastroenteritis. This will have an impact on the norovirus infection. So far, studies had showed that immunosuppressive therapy is a risk factor for norovirus infection [16] and the disease can become chronic and can persist for weeks to years in immunocompromised patients [32]. While in this study, some patients in ward 7 were receiving immunosuppressive therapy because of hematopoietic diseases. Measures for preventing hospital infection were made more carefully in this ward, and this may explain why no significant difference for norovirus positive rates between the two groups in this ward.

The genotype distribution was also analyzed. Results from this study showed that GII.3 and GII.4 noroviruses were the most common genotype in the hospitalized children just as it was found from outpatients in previous study. But the genotype of noroviruses detected from hospitalized children is less diverse than those detected from outpatient. As shown in the phylogenetic tree, the homology of nucleotide sequence is high between most of the strains from the hospitalized children. It further indicates the presence of hospital-acquired infection. At the same time the new variant GII.4 Sydney_2012 has also been detected in 2012 and it had replaced the previous strains stably. Overall, the limitation is that number of cases in this study is small and there may be a bias, more investigation is needed in future. Almost all of the epidemiological studies show that GII.4 norovirus was the predominant genotype identified in presented genotyping data [33,34]. However, in this study, the total detection rate of GII.3 norovirus was reached 50%. Sukhrie FH et al had also mentioned that GII.3 strains were associated with nosocomial spread more often than other viruses in children’s wards, whereas in adults was the GII.4 strains [24]. This may be related to the different evolutionary patterns of the two genotypes. Boon D et al indicated GII.3 noroviruses evolve via selective pressures induced by the host rather than presenting a nucleotide evolution rate lower than that of GII.4 noroviruses[35]. The results may be biased in our study for the small number of specimens, further research is required to investigate this in larger sample studies.

In conclusion, this hospital and laboratory-based investigation for norovirus infection enables to better understand the prevalence of norovirus in hospitalized children with gastroenteritis. The importance of hospital-acquired norovirus infection in children shown in this study indicate that active surveillance of norovirus infection in hospitalized children is necessary to protect the most vulnerable populations.

## Supporting information

S1 Fig Monthly distribution of norovirus-associated acute diarrhea from 2010 to 2013. (a) Monthly distribution of norovirus detection in children from CAI group, CAI: community-acquired infection; (b) Monthly distribution of norovirus detection in children from HAI group, HAI: hospital-acquired infection.
(TIF)

S2 Fig Phylogenetic analysis based on sequences of the partial capsids of the norovirus strains detected in samples from CAI group and HAI group children. Norovirus strains are color-coded as follows: purple, GII.3; green, GII.4 Den Haag_2006b; blue, GII.4 New Orleans_2009; red, GII.4 Sydney_2012; blank, GII.13. Abbreviations of strains: C, community-acquired infection; H, hospital-acquired infection; W, ward.
(TIF)

S1 Table Distribution of norovirus-positive samples detected in Beijing between HAI group and CAI group, 2010–2013. (DOCX)

S2 Table Genotype distribution of norovirus-positive samples. (DOCX)

## References

[1] Donaldson EF, Lindesmith LC, Lobue AD, Baric RS. Norovirus pathogenesis: mechanisms of persistence and immune evasion in human populations. Immunological reviews. 2008;225:190–211. doi: 10.1111/j.1600-065X.2008.00680.x . 18837783 10.1111/j.1600-065X.2008.00680.x

[2] Tran A, Talmud D, Lejeune B, Jovenin N, Renois F, Payan C, et al Prevalence of rotavirus, adenovirus, norovirus, and astrovirus infections and coinfections among hospitalized children in northern France. Journal of clinical microbiology. 2010;48(5):1943–6. doi: 10.1128/JCM.02181-09 ; PubMed Central PMCID: PMC2863921. 20305010 10.1128/JCM.02181-09 PMC2863921

[3] Kapikian AZ. The discovery of the 27-nm Norwalk virus: an historic perspective. The Journal of infectious diseases. 2000;181 Suppl 2:S295–302. doi: 10.1086/315584 . 10804141 10.1086/315584 PMC7110248

[4] Green KY, Chanock RM, Kapikian AZ. Caliciviridae: the noroviruses In Knipe D. M., Howley P. M., Griffin D. E., Lamb R. A., Martin M. A., Roizman B., and Straus S. E. (ed.), Fields virology, 5th ed. Lippincott Williams & Wilkins, Philadelphia, PA 2001:p. 949–979.

[5] Payne DC, Vinje J, Szilagyi PG, Edwards KM, Staat MA, Weinberg GA, et al Norovirus and medically attended gastroenteritis in U.S. children. The New England journal of medicine. 2013;368(12):1121–30. doi: 10.1056/NEJMsa1206589 . 23514289 10.1056/NEJMsa1206589 PMC4618551

[6] Hall AJ, Lopman BA, Payne DC, Patel MM, Gastanaduy PA, Vinje J, et al Norovirus disease in the United States. Emerging infectious diseases. 2013;19(8):1198–205. doi: 10.3201/eid1908.130465 ; PubMed Central PMCID: PMC3739528. 23876403 10.3201/eid1908.130465 PMC3739528

[7] Rydell GE, Kindberg E, Larson G, Svensson L. Susceptibility to winter vomiting disease: a sweet matter. Reviews in medical virology. 2011;21(6):370–82. doi: 10.1002/rmv.704 . 22025362 10.1002/rmv.704

[8] Esposito S, Daleno C, Scala A, Senatore L, Ascolese B, Principi N. Detection of norovirus in respiratory secretions in children with respiratory tract infection. The Pediatric infectious disease journal. 2014;33(3):314–6. doi: 10.1097/INF.0000000000000035 . 23995593 10.1097/INF.0000000000000035

[9] Solano R, Alseda M, Godoy P, Sanz M, Bartolome R, Manzanares-Laya S, et al Person-to-person transmission of norovirus resulting in an outbreak of acute gastroenteritis at a summer camp. European journal of gastroenterology & hepatology. 2014;26(10):1160–6. doi: 10.1097/MEG.0000000000000179 . 25117826 10.1097/MEG.0000000000000179

[10] Hutson AM, Atmar RL, Estes MK. Norovirus disease: changing epidemiology and host susceptibility factors. Trends in microbiology. 2004;12(6):279–87. doi: 10.1016/j.tim.2004.04.005 . 15165606 10.1016/j.tim.2004.04.005 PMC7172956

[11] Atmar RL, Opekun AR, Gilger MA, Estes MK, Crawford SE, Neill FH, et al Norwalk virus shedding after experimental human infection. Emerging infectious diseases. 2008;14(10):1553–7. doi: 10.3201/eid1410.080117 ; PubMed Central PMCID: PMC2609865. 18826818 10.3201/eid1410.080117 PMC2609865

[12] Kaplan JE, Feldman R, Campbell DS, Lookabaugh C, Gary GW. The frequency of a Norwalk-like pattern of illness in outbreaks of acute gastroenteritis. American journal of public health. 1982;72(12):1329–32. ; PubMed Central PMCID: PMC1650540. 6291414 10.2105/ajph.72.12.1329 PMC1650540

[13] Bucardo F, Nordgren J, Carlsson B, Paniagua M, Lindgren PE, Espinoza F, et al Pediatric norovirus diarrhea in Nicaragua. Journal of clinical microbiology. 2008;46(8):2573–80. doi: 10.1128/JCM.00505-08 ; PubMed Central PMCID: PMC2519475. 18562593 10.1128/JCM.00505-08 PMC2519475

[14] Li CS, Chan PK, Tang JW. Prevalence of diarrhea viruses in hospitalized children in Hong Kong in 2008. Journal of medical virology. 2009;81(11):1903–11. doi: 10.1002/jmv.21611 . 19774686 10.1002/jmv.21611

[15] Lorrot M, Bon F, El Hajje MJ, Aho S, Wolfer M, Giraudon H, et al Epidemiology and clinical features of gastroenteritis in hospitalised children: prospective survey during a 2-year period in a Parisian hospital, France. European journal of clinical microbiology & infectious diseases: official publication of the European Society of Clinical Microbiology. 2011;30(3):361–8. doi: 10.1007/s10096-010-1094-9 . 21128089 10.1007/s10096-010-1094-9

[16] Munir N, Liu P, Gastanaduy P, Montes J, Shane A, Moe C. Norovirus infection in immunocompromised children and children with hospital-acquired acute gastroenteritis. Journal of medical virology. 2014;86(7):1203–9. doi: 10.1002/jmv.23774 . 24115094 10.1002/jmv.23774

[17] Zintz C, Bok K, Parada E, Barnes-Eley M, Berke T, Staat MA, et al Prevalence and genetic characterization of caliciviruses among children hospitalized for acute gastroenteritis in the United States. Infection, genetics and evolution: journal of molecular epidemiology and evolutionary genetics in infectious diseases. 2005;5(3):281–90. doi: 10.1016/j.meegid.2004.06.010 . 15737920 10.1016/j.meegid.2004.06.010

[18] Cunliffe NA, Booth JA, Elliot C, Lowe SJ, Sopwith W, Kitchin N, et al Healthcare-associated viral gastroenteritis among children in a large pediatric hospital, United Kingdom. Emerging infectious diseases. 2010;16(1):55–62. doi: 10.3201/eid1601.090401 ; PubMed Central PMCID: PMC2874353. 20031043 10.3201/eid1601.090401 PMC2874353

[19] Liu L, Qian Y, Zhang Y, Deng J, Jia L, Dong H. Adenoviruses associated with acute diarrhea in children in Beijing, China. PloS one. 2014;9(2):e88791 doi: 10.1371/journal.pone.0088791 ; PubMed Central PMCID: PMC3923065. 24533149 10.1371/journal.pone.0088791 PMC3923065

[20] Kojima S, Kageyama T, Fukushi S, Hoshino FB, Shinohara M, Uchida K, et al Genogroup-specific PCR primers for detection of Norwalk-like viruses. Journal of virological methods. 2002;100(1–2):107–14. . 11742657 10.1016/s0166-0934(01)00404-9

[21] Kroneman A, Vennema H, Deforche K, v d Avoort H, Penaranda S, Oberste MS, et al An automated genotyping tool for enteroviruses and noroviruses. Journal of clinical virology: the official publication of the Pan American Society for Clinical Virology. 2011;51(2):121–5. doi: 10.1016/j.jcv.2011.03.006 . 21514213 10.1016/j.jcv.2011.03.006

[22] Beersma MF, Schutten M, Vennema H, Hartwig NG, Mes TH, Osterhaus AD, et al Norovirus in a Dutch tertiary care hospital (2002–2007): frequent nosocomial transmission and dominance of GIIb strains in young children. The Journal of hospital infection. 2009;71(3):199–205. doi: 10.1016/j.jhin.2008.11.018 . 19147255 10.1016/j.jhin.2008.11.018

[23] Franck KT, Nielsen RT, Holzknecht BJ, Ersboll AK, Fischer TK, Bottiger B.Norovirus Genotypes in Hospital Settings: Differences Between Nosocomial and Community-Acquired Infections. The Journal of infectious diseases. 2015;212(6):881–8. doi: 10.1093/infdis/jiv105 . 25701867 10.1093/infdis/jiv105

[24] Sukhrie FH, Beersma MF, Wong A, van der Veer B, Vennema H, Bogerman J, et al Using molecular epidemiology to trace transmission of nosocomial norovirus infection. Journal of clinical microbiology. 2011;49(2):602–6. doi: 10.1128/JCM.01443-10 ; PubMed Central PMCID: PMC3043516. 21159934 10.1128/JCM.01443-10 PMC3043516

[25] Kesson AM, Benwell N, Elliott EJ. Norovirus diarrhoeal disease in infants and children. The Medical journal of Australia. 2010;192(2):108–9. . 20078416 10.5694/j.1326-5377.2010.tb03434.x

[26] Spackova M, Altmann D, Eckmanns T, Koch J, Krause G. High level of gastrointestinal nosocomial infections in the german surveillance system, 2002–2008. Infection control and hospital epidemiology. 2010;31(12):1273–8. doi: 10.1086/657133 . 21047180 10.1086/657133

[27] Gonzalez-Galan V, Sanchez-Fauqier A, Obando I, Montero V, Fernandez M, Torres MJ, et al High prevalence of community-acquired norovirus gastroenteritis among hospitalized children: a prospective study. Clinical microbiology and infection: the official publication of the European Society of Clinical Microbiology and Infectious Diseases. 2011;17(12):1895–9. doi: 10.1111/j.1469-0691.2011.03506.x . 21848976 10.1111/j.1469-0691.2011.03506.x

[28] Rasanen S, Lappalainen S, Salminen M, Huhti L, Vesikari T. Noroviruses in children seen in a hospital for acute gastroenteritis in Finland. European journal of pediatrics. 2011;170(11):1413–8. doi: 10.1007/s00431-011-1443-4 ; PubMed Central PMCID: PMC3197931. 21465124 10.1007/s00431-011-1443-4 PMC3197931

[29] Medici MC, Martinelli M, Abelli LA, Ruggeri FM, Di Bartolo I, Arcangeletti MC, et al Molecular epidemiology of norovirus infections in sporadic cases of viral gastroenteritis among children in Northern Italy. Journal of medical virology. 2006;78(11):1486–92. doi: 10.1002/jmv.20723 . 16998898 10.1002/jmv.20723

[30] Jia LP, Qian Y, Zhang Y, Deng L, Liu LY, Zhu RN, et al Prevalence and genetic diversity of noroviruses in outpatient pediatric clinics in Beijing, China 2010–2012. Infection, genetics and evolution: journal of molecular epidemiology and evolutionary genetics in infectious diseases. 2014;28:71–7. doi: 10.1016/j.meegid.2014.09.006 . 25218087 10.1016/j.meegid.2014.09.006

[31] Green J, Wright PA, Gallimore CI, Mitchell O, Morgan-Capner P, Brown DW. The role of environmental contamination with small round structured viruses in a hospital outbreak investigated by reverse-transcriptase polymerase chain reaction assay. The Journal of hospital infection. 1998;39(1):39–45. . 9617683 10.1016/s0195-6701(98)90241-9

[32] Sukhrie FH, Teunis P, Vennema H, Copra C, Thijs Beersma MF, Bogerman J, et al Nosocomial transmission of norovirus is mainly caused by symptomatic cases. Clinical infectious diseases: an official publication of the Infectious Diseases Society of America. 2012;54(7):931–7. doi: 10.1093/cid/cir971 . 22291099 10.1093/cid/cir971

[33] Bernard H, Hohne M, Niendorf S, Altmann D, Stark K. Epidemiology of norovirus gastroenteritis in Germany 2001–2009: eight seasons of routine surveillance. Epidemiology and infection. 2014;142(1):63–74. doi: 10.1017/S0950268813000435 . 23517686 10.1017/S0950268813000435 PMC9152553

[34] Tsai CN, Lin CY, Lin CW, Shih KC, Chiu CH, Chen SY. Clinical relevance and genotypes of circulating noroviruses in northern Taiwan, 2006–2011. Journal of medical virology. 2014;86(2):335–46. doi: 10.1002/jmv.23728 . 24009100 10.1002/jmv.23728

[35] Boon D, Mahar JE, Abente EJ, Kirkwood CD, Purcell RH, Kapikian AZ, et al Comparative evolution of GII.3 and GII.4 norovirus over a 31-year period. Journal of virology. 2011;85(17):8656–66. doi: 10.1128/JVI.00472-11 ; PubMed Central PMCID: PMC3165818. 21715504 10.1128/JVI.00472-11 PMC3165818

