# Supplementary figures and images for: High prevalence of GII norovirus in hospitalized children with acute diarrhea, in Beijing
Source: PLoS One. 2017 Jun 29;12(6):e0179839. doi: 10.1371/journal.pone.0179839 (PMC5491042; doi:10.1371/journal.pone.0179839)

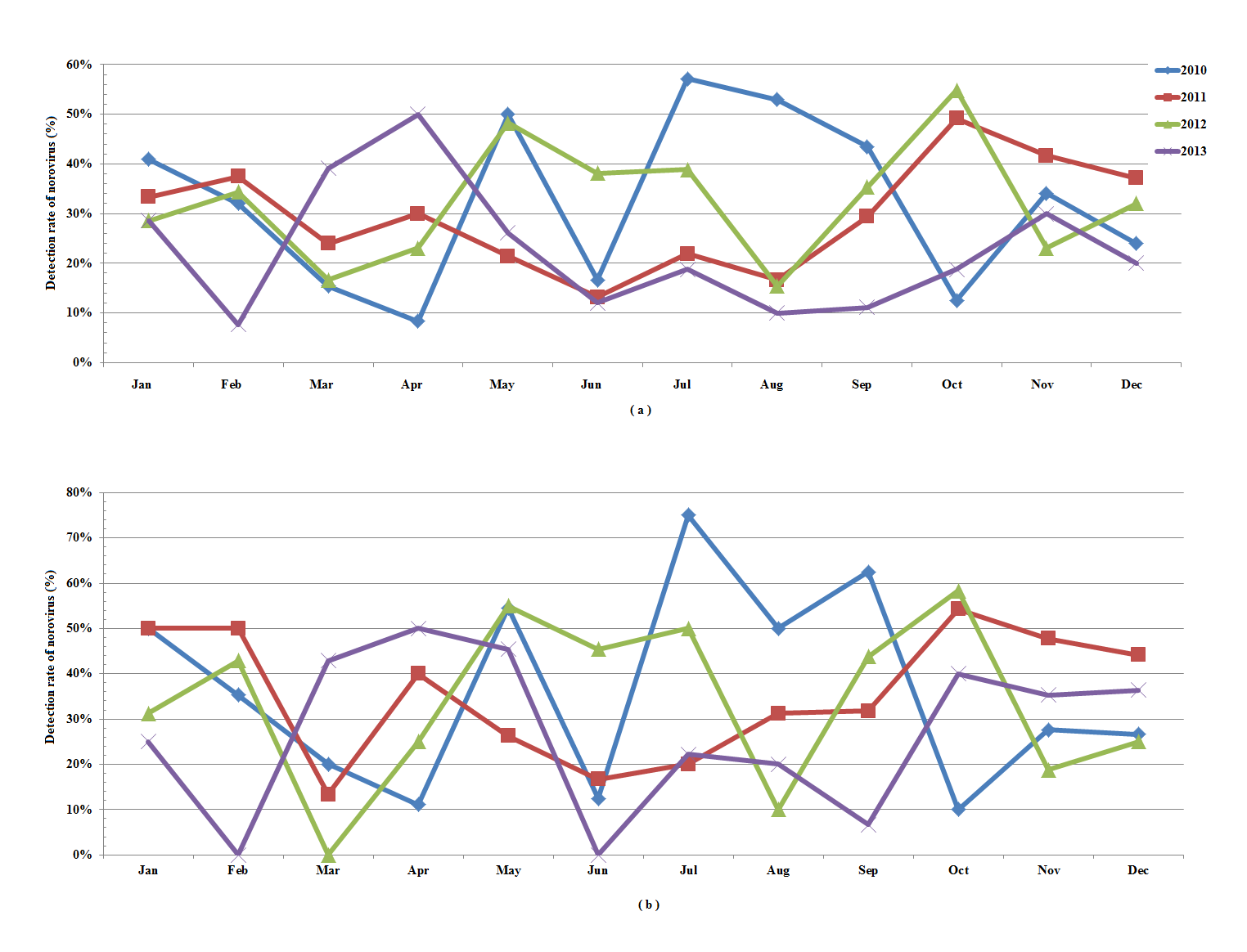

Supplement: S1 Fig — (a) Monthly distribution of norovirus detection in children from CAI group, CAI: community-acquired infection; (b) Monthly distribution of norovirus detection in children from HAI group, HAI: hospital-acquired infection. (TIF) [file pone.0179839.s001.tif]

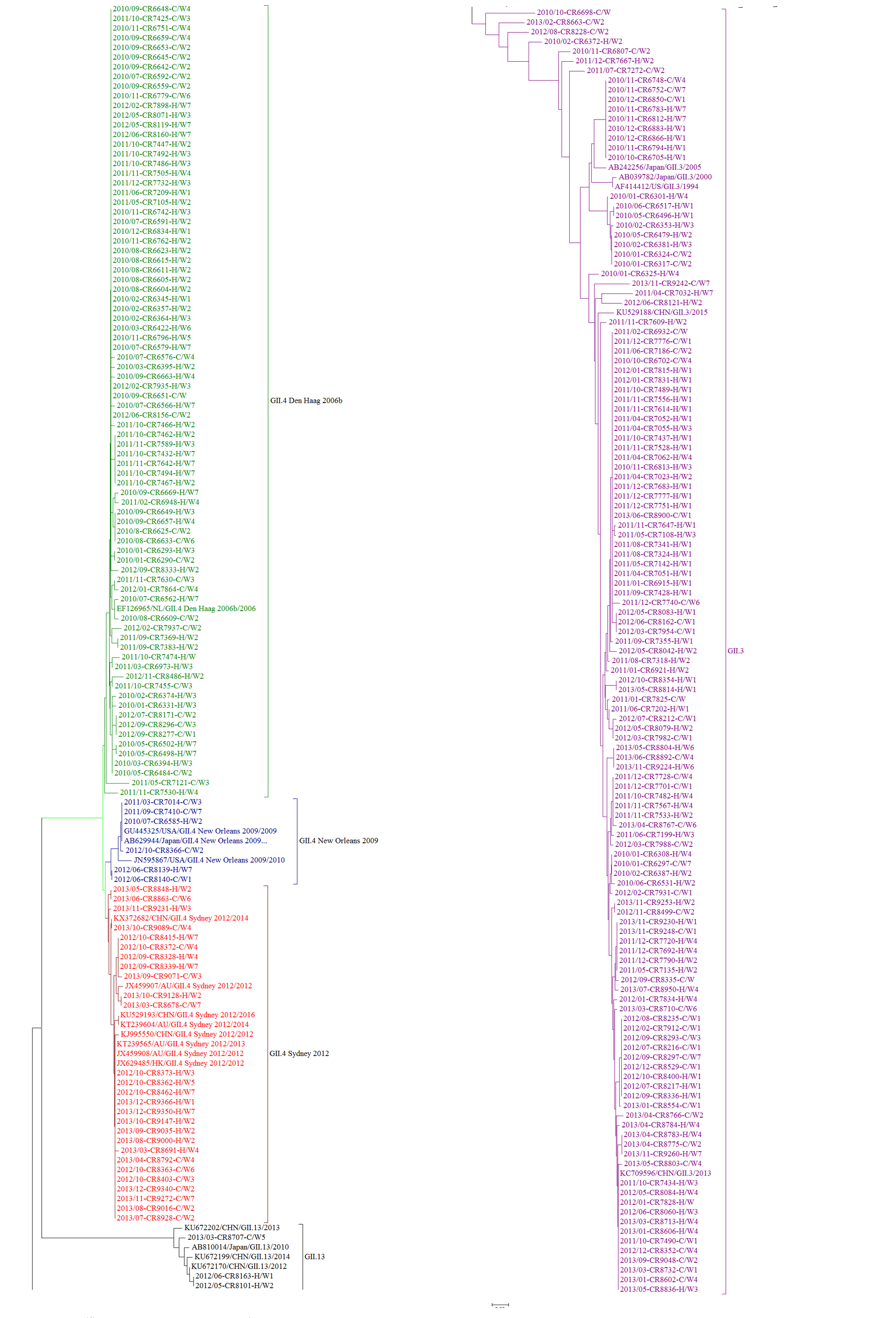

Supplement: S2 Fig — Norovirus strains are color-coded as follows: purple, GII.3; green, GII.4 Den Haag_2006b; blue, GII.4 New Orleans_2009; red, GII.4 Sydney_2012; blank, GII.13. Abbreviations of strains: C, community-acquired infection; H, hospital-acquired infection; W, ward. (TIF) [file pone.0179839.s002.tif]
