# Supplementary material for: High prevalence of GII norovirus in hospitalized children with acute diarrhea, in Beijing
Source: PLoS One. 2017 Jun 29;12(6):e0179839. doi: 10.1371/journal.pone.0179839 (PMC5491042; doi:10.1371/journal.pone.0179839)
Supplement: S1 Table — (DOCX) [file pone.0179839.s003.docx]

**Table S1. Distribution of Norovirus-positive samples detected in Beijing between HAI group and CAI group, 2010-2013**

| **Distributions** | **HAI group (No.)** | | **CAI group (No.)** | | ***X^2^*** | ***P*** |
| --- | --- | --- | --- | --- | --- | --- |
|  | **Total** | **No. of NoV positives (%)** | **Total** | **No. of NoV positives (%)** |  |  |
| **Total** | 661 | 232(35.1) | 587 | 148(25.2) | 14.35 | 0.001 |
| **Year of collection** |  |  |  |  |  |  |
| 2010 | 150 | 51(34.0) | 108 | 31(28.7) | 0.81 | 0.367 |
| 2011 | 269 | 101(37.6) | 179 | 42(23.5) | 9.81 | 0.002 |
| 2012 | 141 | 52(36.9) | 160 | 49(30.6) | 1.32 | 0.252 |
| 2013 | 101 | 28(27.7) | 140 | 26(18.6) | 2.83 | 0.093 |
| **Age distributions of Girl** |  |  |  |  |  |  |
| 0-5 month | 82 | 33(40.2) | 108 | 20(18.5) | 10.94 | 0.001 |
| 6-11month | 41 | 13(31.7) | 56 | 16(28.6) | 0.11 | 0.739 |
| 12-23 month | 49 | 14(28.6) | 48 | 15(31.3) | 0.08 | 0.773 |
| 24-35 month | 24 | 7(29.2) | 10 | 0(0.00) | 3.67 | 0.055 |
| ≥36 month | 23 | 4(17.4) | 12 | 1(8.3) | 0.53 | 0.467 |
|  | *X^2^=5.12 p=0.275* | | *X^2^=8.62 p=0.071* | |  |  |
| total | 219 | 71(32.4) | 234 | 52(22.2) | 5.95 | 0.015 |
| **Age distributions of Boy** |  |  |  |  |  |  |
| 0-5 month | 154 | 54(35.1) | 147 | 34(23.1) | 5.18 | 0.023 |
| 6-11month | 101 | 40(39.6) | 88 | 25(28.4) | 2.61 | 0.106 |
| 12-23 month | 122 | 56(45.9) | 83 | 33(39.8) | 0.76 | 0.384 |
| 24-35 month | 26 | 6(23.1) | 14 | 1(7.1) | 1.4 | 0.206 |
| ≥36 month | 40 | 5(12.5) | 20 | 3(15.0) | 0.02 | 0.788 |
|  | *X^2^=17.20 p=0.02* | | *X^2^=12.23 p=0.016* | |  |  |
| total | 443 | 161(36.3) | 352 | 96(27.3) | 7.38 | 0.007 |
| **Wards distributions** |  |  |  |  |  |  |
| Ward 1 | 119 | 52(43.7) | 130 | 33(25.4) | 9.27 | 0.002 |
| Ward 2 | 136 | 53(39.0) | 191 | 45(23.5) | 8.99 | 0.003 |
| Ward 3 | 100 | 42(42.0) | 74 | 21(28.4) | 3.42 | 0.065 |
| Ward 4 | 111 | 40(36.0) | 95 | 26(27.4) | 1.77 | 0.184 |
| Ward 5 and Ward 6 | 70 | 10(14.3) | 68 | 15(22.1) | 1.40 | 0.236 |
| Ward 7 | 125 | 35(28.0) | 29 | 8(27.6) | 0.01 | 0.964 |

Abbreviations: HAI, hospital-acquired infection; CAI, community-acquired infection; No., Numbers of specimens; (%) Positive = Positivity of Norovirus.
