# Supplementary material for: High prevalence of GII norovirus in hospitalized children with acute diarrhea, in Beijing
Source: PLoS One. 2017 Jun 29;12(6):e0179839. doi: 10.1371/journal.pone.0179839 (PMC5491042; doi:10.1371/journal.pone.0179839)
Supplement: S2 Table — (DOCX) [file pone.0179839.s004.docx]

**Table S2. Genotype distribution of Norovirus-positive samples**

|  | HAI group (No.) | | | | | | CAI group (No.) | | | | | |
| --- | --- | --- | --- | --- | --- | --- | --- | --- | --- | --- | --- | --- |
|  | GII.3 | GII.4^a^ | GII.4^b^ | GII.4^c^ | GII.13 | Total^d^ | GII.3 | GII.4a | GII.4b | GII.4c | GII.13 | Total^d^ |
| **Years of collection** |  |  |  |  |  |  |  |  |  |  |  |  |
| 2010 | 18 | 28 | 1 | 0 | 0 | 150 | 9 | 17 | 0 | 0 | 0 | 108 |
| 2011 | 40 | 20 | 0 | 0 | 0 | 269 | 8 | 10 | 3 | 0 | 0 | 179 |
| 2012 | 14 | 10 | 0 | 6 | 2 | 141 | 17 | 6 | 2 | 5 | 0 | 160 |
| 2013 | 13 | 0 | 0 | 11 | 0 | 101 | 12 | 0 | 0 | 9 | 1 | 140 |
| **Wards distributions** |  |  |  |  |  |  |  |  |  |  |  |  |
| Ward 1 | 36 | 3 | 0 | 1 | 2 | 119 | 19 | 1 | 1 | 0 | 0 | 130 |
| Ward 2 | 17 | 21 | 1 | 5 | 0 | 136 | 13 | 15 | 2 | 4 | 0 | 191 |
| Ward 3 | 8 | 14 | 0 | 3 | 0 | 100 | 1 | 6 | 1 | 2 | 0 | 74 |
| Ward 4 | 16 | 5 | 0 | 2 | 0 | 111 | 6 | 6 | 0 | 4 | 0 | 95 |
| Ward 5 and Ward 6 | 2 | 2 | 0 | 1 | 0 | 70 | 3 | 5 | 0 | 2 | 1 | 68 |
| Ward 7 | 6 | 13 | 0 | 5 | 0 | 125 | 4 | 0 | 1 | 2 | 0 | 29 |

Abbreviations: HAI, hospital-acquired infection; CAI, community-acquired infection; No., Numbers of specimens.

^a^ GII.4 Den Haag_2006 norovirus.

^b^ GII.4 New Orleans_2009 norovirus.

^c^ GII.4Sydney_2012 norovirus.

^d^ Total numbers of enrolled specimens.
